# Supplementary material for: Disentangling metabolic functions of bacteria in the honey bee gut
Source: PLoS Biol. 2017 Dec 12;15(12):e2003467. doi: 10.1371/journal.pbio.2003467 (PMC5726620; doi:10.1371/journal.pbio.2003467)

Heliotrine

619 312.181 microbial substrate

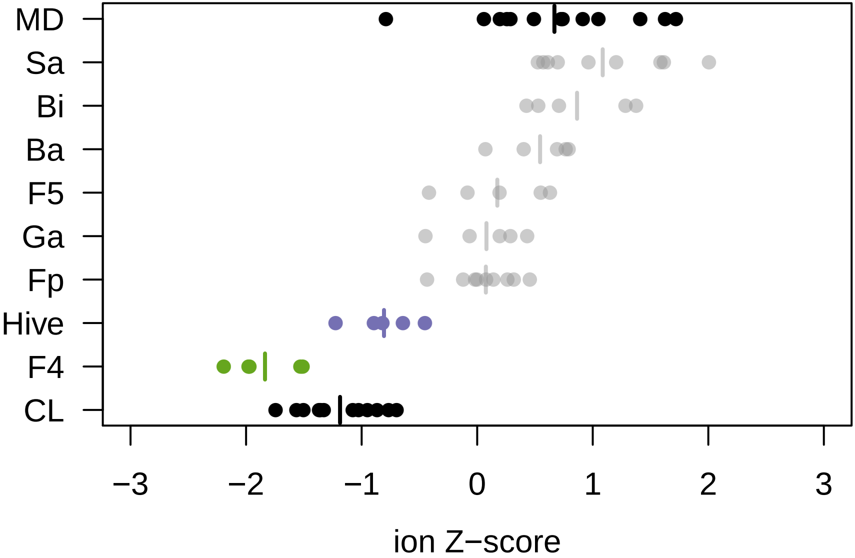

Supplement: S8 Data — (ZIP) [file pbio.2003467.s008.zip › Z-score_plots/619 microbial substrate Heliotrine.pdf]
